# Adaptable and comprehensive approaches for long-read nanopore sequencing of polyadenylated and non-polyadenylated RNAs

**DOI:** 10.3389/fgene.2024.1466338

**Published:** 2024-12-02

**Authors:** Simon Haile, Richard D. Corbett, Kieran O’Neill, Jing Xu, Duane E. Smailus, Pawan K. Pandoh, Anthony Bayega, Miruna Bala, Eric Chuah, Robin J. N. Coope, Richard A. Moore, Karen L. Mungall, Yongjun Zhao, Yussanne Ma, Marco A. Marra, Steven J. M. Jones, Andrew J. Mungall

**Affiliations:** ^1^ Canada’s Michael Smith Genome Sciences Centre, BC Cancer, Vancouver, BC, Canada; ^2^ Department of Medical Genetics, University of British Columbia, Vancouver, BC, Canada

**Keywords:** RNA-seq, Oxford Nanopore, cDNA, full-length, polyadenylated and non-polyadenylated, long-read, transcriptome, Ribosomal RNA depletion

## Abstract

The advent of long-read (LR) sequencing technologies has provided a direct opportunity to determine the structure of transcripts with potential for end-to-end sequencing of full-length RNAs. LR methods that have been described to date include commercial offerings from Oxford Nanopore Technologies (ONT) and Pacific Biosciences. These kits are based on selection of polyadenylated (polyA+) RNAs and/or oligo-dT priming of reverse transcription. Thus, these approaches do not allow comprehensive interrogation of the transcriptome due to their exclusion of non-polyadenylated (polyA-) RNAs. In addition, polyA + specificity also results in 3′-biased measurements of PolyA+ RNAs especially when the RNA input is partially degraded. To address these limitations of current LR protocols, we modified rRNA depletion protocols that have been used in short-read sequencing: one approach representing a ligation-based method and the other a template-switch cDNA synthesis-based method to append ONT-specific adaptor sequences and by removing any deliberate fragmentation/shearing of RNA/cDNA. Here, we present comparisons with poly+ RNA-specific versions of the two approaches including the ONT PCR-cDNA Barcoding kit. The rRNA depletion protocols displayed higher proportions (30%–50%) of intronic content compared to that of the polyA-specific protocols (5%–8%). In addition, the rRNA depletion protocols enabled ∼20–50% higher detection of expressed genes. Other metrics that were favourable to the rRNA depletion protocols include better coverage of long transcripts, and higher accuracy and reproducibility of expression measurements. Overall, these results indicate that the rRNA depletion-based protocols described here allow the comprehensive characterization of polyadenylated and non-polyadenylated RNAs. While the resulting reads are long enough to help decipher transcript structures, future endeavors are warranted to improve the proportion of individual reads representing end-to-end spanning of transcripts.

## Introduction

Whereas short-read sequencing platforms continue to be powerful in transcriptome characterization and quantification, they rely on computational assembly tools to generate contiguous sequences from short reads and thereby extrapolate on transcript structural variations such as alternate transcription start sites, splice isoforms, alternative polyadenylation and fusion transcripts. Linked-read technologies (Tilgner et al., 2015; Tilgner et al., 2018) have partially improved such assessments but more significant improvements have been made with the advent of long-read (LR) technologies from Pacific Biosciences (PacBio) (Sharon et al., 2013) and Oxford Nanopore Technologies (ONT) (Oikonomopoulos et al., 2016). LR platforms have provided an opportunity to directly determine the structure of transcripts including the possibility of end-to-end sequencing of full-length RNAs.

Ribosomal RNAs (rRNAs) represent >90% of the total RNA mass within cells (Lucas et al., 1977; Sturani et al., 1979) thereby limiting the sensitivity of RNA-seq to detect mRNA transcripts. Several methods that either enrich for mRNAs or deplete rRNAs have been developed. Capture of non-rRNA transcripts can be attained by targeting their poly(A) tails (Aviv and Leder, 1972), as most rRNAs are not polyadenylated (Kuai et al., 2004). However, poly(A) enrichment strategies can result in a strong bias towards recovery of only the 3′-ends of transcripts if the input RNA samples are degraded. Alternatively, methods that specifically remove rRNAs can be targeted (Adiconis et al., 2013; Morlan et al., 2012; Hrdlickova et al., 2017). These protocols also allow the assessment of non-polyadenylated mRNAs and they include the affinity purification-based Ribo-Zero Gold kit (Illumina) (Adiconis et al., 2013) and the enzyme-based New England Biolabs (NEB) protocol (Morlan et al., 2012) that work at the RNA level. Alternatively, protocols that use enzymatic probe-directed degradation approach at the cDNA level have been reported (Archer et al., 2014; Verboom et al., 2019).

The majority of existing LR methods, including commercial offerings, are based on selection of polyadenylated mRNAs (polyA+ RNAs) and/or oligo-dT priming of reverse transcription (Soneson C et al., 2019). Thus, these approaches do not allow comprehensive interrogation of the transcriptome due to their exclusion of non-polyadenylated RNAs (polyA- RNAs). In addition, polyA+ specificity also results in 3′-biased measurements of polyA+ RNAs when the RNA input is partially degraded. An exception is an approach that is based on the (PCR-free) direct RNA ONT sequencing kit but its application is limited to mostly cell line-derived RNA as the input requirement for the protocol is >100 μg (Ibrahim et al., 2021).

To address the aforementioned limitations of current LR protocols, we modified two ribosomal RNA (rRNA) depletion and cDNA-based protocols that have been employed in short-read sequencing and adapted them for ONT sequencing. One of these represents a cDNA ligation-based method using the NEB’s rRNA depletion kit at the RNA level (Morlan et al., 2012; Haile S et al., 2019) and the other involves a template-switch cDNA synthesis method that employs rRNA depletion at the cDNA level (Verboom et al., 2019; Haile S et al., 2021). Here, we compare these protocols with polyA+ RNA-specific versions of the two approaches including the commercially available ONT PCR-cDNA barcoding kit for the method that is based on template-switch cDNA synthesis.

## Methods and materials

### Samples

Universal Human Reference (UHR) total RNA (Stratagene catalog #740000) was quantified using the RNA 6000 Nano Kit (Agilent, catalog #5067–1511). The SIRV-Set four synthetic RNA spiking mix (Lexogen, catalog # 141) was added to UHR total RNA to allow for assessments of expression accuracy, sensitivity, and transcript isoform detection. A 1.12 μL volume of the spike-in mix stock was used per 10 μg UHR total RNA (this is equivalent to ∼6 pg and ∼120 pg of the spike-in RNA mix per the 10 ng and 200 ng total RNA input amounts, respectively).

### Library construction

#### Modified RNase H-based rRNA depletion

New England Biolabs’ (NEB) RNase H-based rRNA depletion protocol (cat.no. E6310X, NEB, United States) was applied to 200–1000 ng of DNase-I treated total RNA as previously described (Haile S et al., 2019).

Following rRNA depletion, cDNA synthesis and library construction steps were performed as described (Haile et al., 2021; Haile S et al.,, 2019) with the following modifications: (1) bead-based purifications were modified to allow bead to reaction mix ratio of 1:1 during cDNA synthesis, (2) mechanical shearing of cDNA was omitted, (3) bead-based purification after ligation with ONT specific adaptor was performed using a bead to reaction mix ratio of 0.8:1, (4) Digestion of dUTP containing cDNA strand using Uracil-Specific Excision Reagent (USER) was applied as a separate step after bead-based purification of the ligated product, (5) LongAmp PCR enzyme from NEB was used for PCR enrichment with ONT multiplexing primers that are part of the ONT PCR-barcoding kit (SQK-PCB111.24) and (6) only one bead-based purification was performed after 13 cycles of PCR with a bead to reaction mix ratio of 0.7:1. Detailed step-by-step library construction protocol is described in the supplementary section.

#### Modified SMARTer total RNA library prep

**FIGURE 1:**
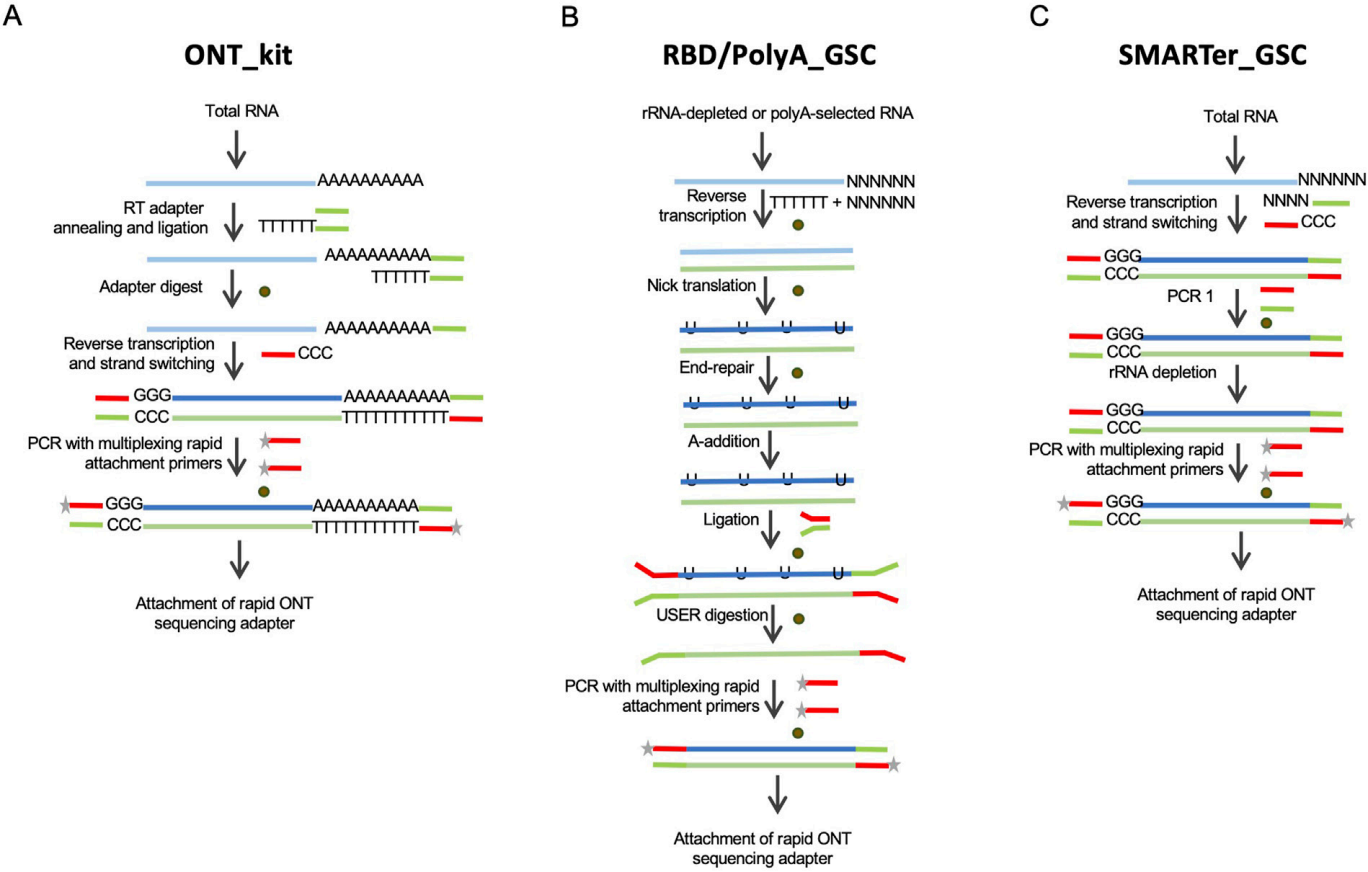
Schematic depictions of the library preparation approaches evaluated in this study. **(A)** The Oxford Nanopore kit, “ONT kit”, is based on cDNA synthesis upon priming at the 3′-end polyadenylation tails of mRNAs and template-switch priming at the 5′-end. **(B)** RNAse H-based rRNA depletion in-house protocol, RBD-GSC, involves ligation of full-length double-stranded cDNA products with ONT-specific adaptors. The protocol involves random priming for first strand cDNA synthesis. A variation of this ligation-based protocol, “GSC-PolyA”, starts with poly(A)-selected mRNAs. **(C)** Modified SMARTer total RNA protocol, “SMARTer-GSC”, involves the removal of rRNA after conversion to cDNA. Brown circles represent magnetic bead-based purifications.

The SMART-Seq^®^ Stranded Kit (Takara Bio; cat no 634444) was used with the following modifications: (1) Universal ONT-compatible primers were used for the first PCR (PCR-1) instead of the indexing primers that come with the SMART-Seq^®^ Stranded Kit; (2) ONT multiplexing primers that are part of the ONT PCR-barcoding kit (SQK-PCB111.24) were used for the second PCR (PCR-2); (3) Five cycles and two cycles of PCR-1 were applied for the 10 ng UHR and 200 ng UHR, respectively. Twelve cycles of PCR-2 were applied to both input amounts. Of note, PCR-1 is light amplification that is applied to bring the cDNA levels to an appreciable narrower range of yield before the rRNA depletion step (Figure 1). The second (PCR-2) is an enrichment step to bring to a library amount sufficient for sequencing while also completing the adapter constructs needed for sequencing and where multiplexing barcodes were also introduced. Bead-based purifications were modified to allow bead to reaction mix ratio of 1:1 before PCR-2 and 0.7:1 following PCR-2.

### Bioinformatic analyses

Base calling of the raw fast5 signal files was performed using Guppy 6.3.7 and the model “dna_r9.4.1_450bps_sup_prom.cfg” after which sequence reads passing default quality filters were stored in fastq format. Reads were reverse complemented when the barcode variant in the sequencing_summary.txt file listed the orientation as “var2”.

Each of the five conditions (four protocols, five input amounts) were randomly down-sampled to 12 million reads. RNA alignment metrics, including 3′-5′ coverage plots, were generated using gatk-4.1.9.0 CollectRNASeqMetrics after aligning the down-sampled reads with Minimap 2.25-r1173 (minimap2 -t 16 -ax splice) to a reference containing contigs from hg38, ERCC, and SIRVs. BamSlam (downloaded in April 2023) was used to collect metrics related to transcript completeness after minimap aligning (minimap2 -ax map-ont--sam-hit-only) the down-sampled reads to transcript models from GenCode42, ERCC and SIRVs. To generate TPM expression estimates, Salmon 1.9.0 (salmon quant -l A--ont -g gencode_gene_transcript.txt--gencode) was applied to the transcript-aligned reads using a.gtf file containing GenCode42, ERCC, and SIRV transcripts.

## Results and discussion

### Protocol development and evaluation overview

Currently, the only commercially available protocol for cDNA sequencing on Oxford Nanopore platforms is based on cDNA synthesis upon priming at the 3′-end polyadenylation tails of mRNAs and template-switch priming at the 5′-end (Figure 1A). This kit (henceforth referred to as “ONT kit”) is, therefore, not suitable for sequencing non-polyadenylated RNAs. To address this limitation, we modified an RNase H-based rRNA depletion protocol that we previously applied to short-read sequencing (Haile S et al., 2019) (Figure 1B). This protocol (“RBD_GSC”) involves ligation of full-length double-stranded cDNA products with ONT-specific adaptors. Of note, the first strand cDNA synthesis in this protocol involves random priming to be able to capture transcripts comprehensively regardless of their polyadenylation status. A variation of this ligation-based protocol (“PolyA_GSC”) that starts with poly(A)-selected mRNAs is also included for comparison (Figure 1B). The RBD_GSC protocol included significant manipulation of the RNA including high temperature incubations during the rRNA depletion steps which may result in partial degradation. We, therefore, considered another protocol that involves the removal of rRNA after conversion to cDNA with minimal opportunity to degrade RNA. This protocol (“SMARTer-GSC”) is based on the SMART-Seq^®^ Stranded Kit from Takara Bio (Verboom et al., 2019; Haile S et al., 2021) following our modifications to render it ONT compatible (Figure 1C). An added advantage of SMARTer-GSC is that it allows the use of lower input amounts (as low as picograms of total RNA). Besides the addition of ONT adapters, all the modified protocols also avoided any deliberate shearing/fragmentation of RNA/cDNA to capitalise on the long read feature of the ONT platform.

Here, we sought to compare the aforementioned protocols using Universal Human Reference (UHR) total RNA as input. cDNA libraries were made from 200 ng total RNA for all protocols. We also included a 10 ng input amount for the SMARTer-GSC protocol. Libraries were made in triplicate for each protocol and two randomly selected replicates from each protocol were pooled for sequencing on one PromethION flowcell. After seeing reproducible results from replicates at shallower sequencing level (data not shown), reads from replicates for each of the protocols/input amounts were merged and randomly down-sampled to 12 million for normalization of sequencing depth.

### Read length distributions

**FIGURE 2:**
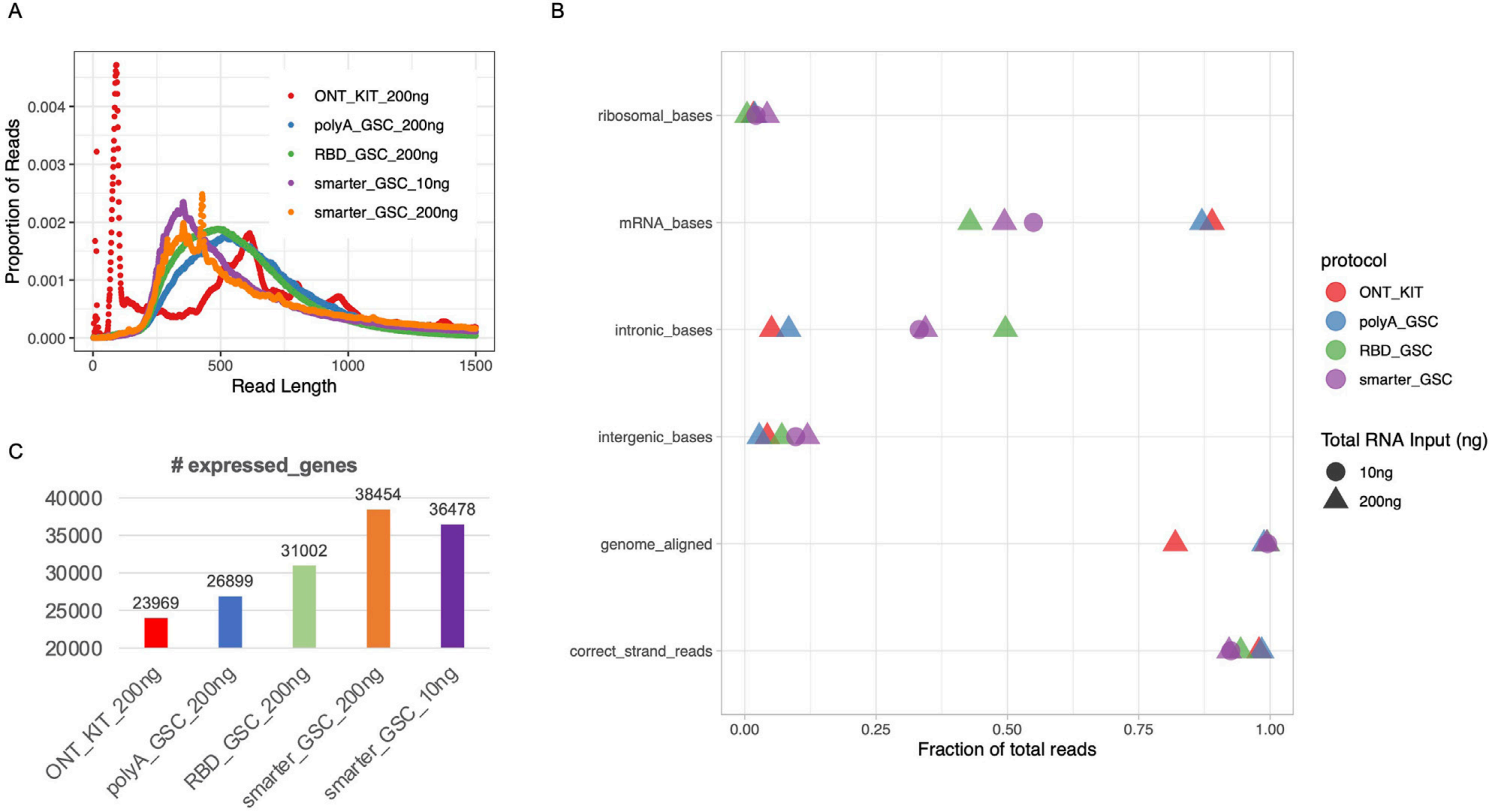
Read length distribution and gene-level metrics. **(A)** Read length distribution upon sequencing of libraries that were generated using the various protocols. **(B)** Various alignment-based quality metrics. **(C)** Number of expressed genes. All libraries were generated from 200 ng total Universal Human Reference (UHR) RNA. For “GSC-SMARTer”, libraries were also generated from 10 ng total RNA.

The ONT kit libraries displayed a very high spike of <125 nucleotides (nt) (Figure 2A), the majority of which could not be mapped to the human genome and contain stretches of Ts presumably corresponding to truncated reads largely limited to the polyA tail region of transcripts (data not shown). There were also other spikes of >500 nt (Figure 2A). The 10 ng and 200 ng SMARTer_GSC libraries displayed the highest median read length (3,699 and 4,450 nt, respectively) and the RBD_GSC libraries displayed the lowest (1,634 nt) while the ONT_KIT and PolyA_GSC showed a median length in between the two protocols; 2,278 nt and 2,000 nt, respectively. (Supplementary Figure 1A). The trend in read distribution is consistent with what was observed when the final libraries were size profiled using an Agilent Bioanalyzer (Supplementary Figure 1B). However, there appears to be higher proportion of smaller fragments upon ONT sequencing (Figure 2A) compared to the proportion of smaller fragments observed using the Bioanalyzer assessment reflecting the sequencing bias towards smaller fragments and/or inaccuracy of Bioanalyzer size profiling (Supplementary Figure 1D).

### Genome-level post-alignment metrics and sensitivity of detection of expressed genes

The proportion of reads that mapped to the human genome was high (>98%) for all protocols except the ONT-kit (∼82%) (Figure 2B), which in part might be due to the polyA tail mapping artifacts mentioned above. Residual rRNA levels were low for all protocols (<5%) with the RBD-GSC protocol displaying the lowest (∼0.4%) compared to the others (1.6%–4.2%) (Figure 2B).

As expected, the two rRNA depletion protocols (SMARTer-GSC and RBD-GSC) yielded higher proportions (30%–50%) of reads mapping to intronic regions compared to the two polyA-specific protocols (PolyA_GSC and ONT-kit) which displayed 5%–8% intronic content (Figure 2B). This trend is consistent with what we previously observed from similar UHR libraries that were sequenced on the Illumina platform (Haile et al., 2021; Haile S et al., 2019) and reflects the capacity of the rRNA depletion-based protocols to capture non-polyadenylated RNAs.

Strand-specificity was high for all protocols with >92% of reads mapping to the expected strand with the rRNA depletion protocols showing lower strand-specificity (92%–94%) compared to the polyA-specific protocols (∼98%) (Figure 2B); perhaps due to higher representation of unannotated anti-sense non-polyadenylated transcripts in the former.

Sensitivity of detection of expressed genes was measured by counting the number of genes with >0 read counts. This analysis demonstrated that the ONT_kit displayed the lowest number of expressed genes (∼24,000) and the PolyA_GSC displayed the next highest number of genes (∼27,000). RBD_GSC and SMARTer_GSC protocols exhibited even higher numbers of genes detected (31–38,000) (Figure 2C); consistent with the expectation that the rRNA depletion-based protocols are more comprehensive in detecting polyA and non-polyA transcripts.

### Capacity to represent full-length transcripts and the degree of uniformity of transcript body coverage

**FIGURE 3:**
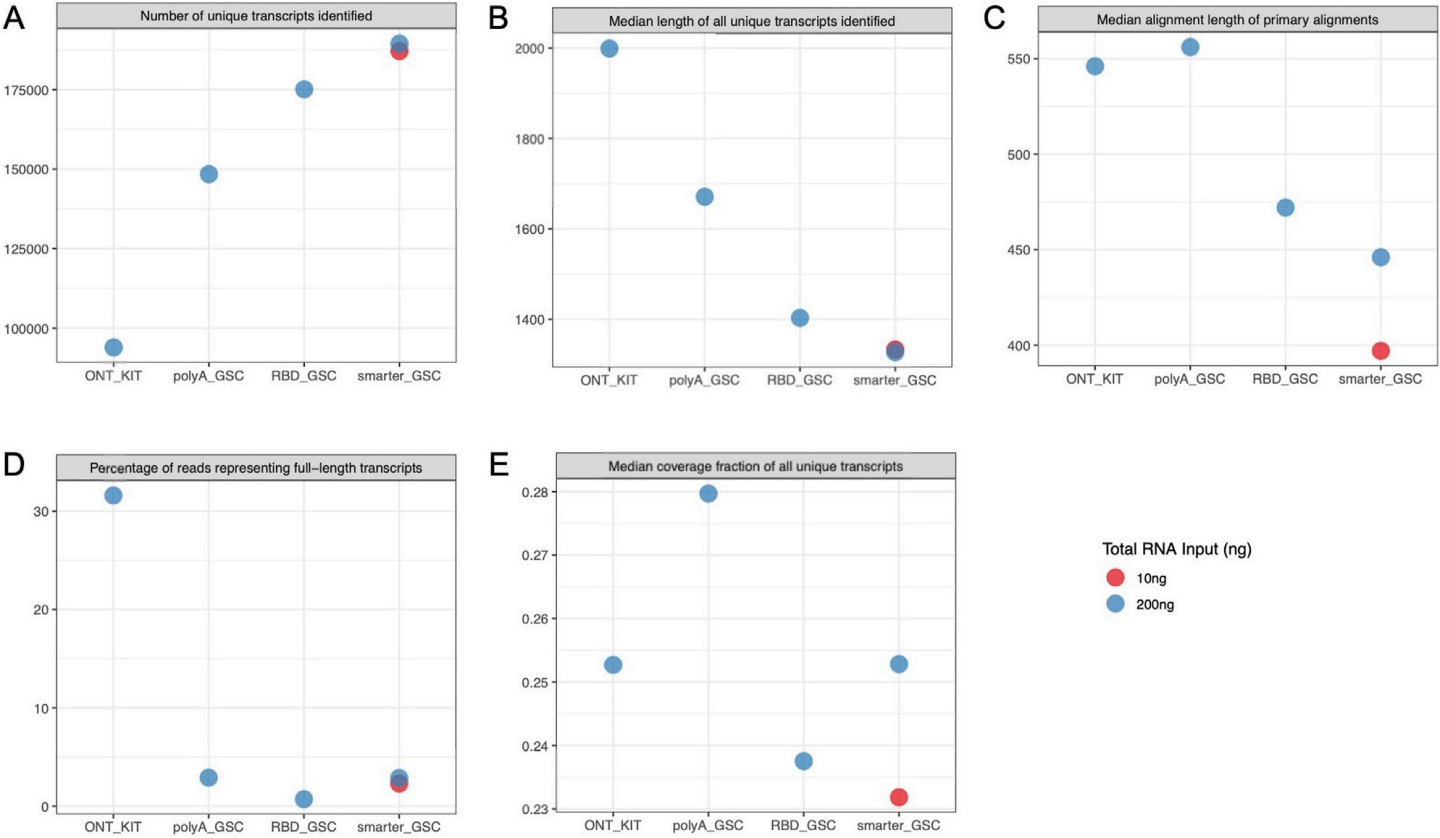
Transcript-level metrics. Various metrics representing sensitivity of detection of transcripts **(A)**, median length of unique transcripts **(B)**, read length of contiguous alignments to transcripts **(C)**, and transcript coverage **(D, E)**. All libraries were generated from 200 ng total Universal Human Reference (UHR) RNA. For “GSC-SMARTer ”, libraries were also generated from 10 ng total RNA.

Transcriptome-level analysis was performed using the BamSlam pipeline that was specifically developed for assessing long-read RNA-seq data (Gleeson J et al., 2022). This analysis showed that the ONT-kit detected the lowest number of unique transcripts (93,917) compared to the other protocols (148,394–189,292) (Figure 3A); consistent with the sensitivity trend observed at the gene level (Figure 2C). Median length of uniquely identified transcripts was highest for the ONT-kit (1,999 nt), followed by PolyA_GSC (1,403 nt), and the lowest for RBD_GSC (1,333 nt) and SMARTer_GSC (1,327 nt) (Figure 3B). This may in part be due to the differences in the length of polyA+ vs polyA-transcripts and the relative abundances of RNAs therein.

The trend of the length of primary alignments (Figure 3C) was consistent with read length distributions (Figure 2A). The BamSlam tool defines reads representing full-length transcripts as those that span 95% of the transcript they primarily align to. The percentage of reads representing full-length transcripts was ∼31.6% for the ONT-kit libraries whereas this metric for the other libraries generated using the other protocols was 0.7%–2.9% (Figure 3D). Visual inspection of IGV read distribution supported this trend (data not shown). Despite this trend, the median coverage fraction for all unique transcripts was comparable for the four protocols, ranging between 0.22 and 0.28 (Figure 3E); indicating that the higher percentage of reads representing full-length transcripts for the ONT kit is skewed towards a limited number of transcripts.

**FIGURE 4:**
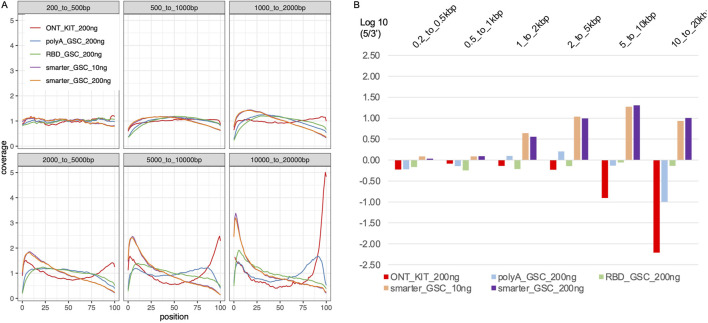
Transcript body coverage. **(A)** Comparison of the normalized coverage of transcript bodies, from 5′(left; 0 in *x*-axis) to 3′(right; 100 in *x*-axis) of all annotated termini between the various protocols. **(B)** Ratio of 5′-end coverage to 3′-end coverage representing distal 100 nt ends. All libraries were generated from 200 ng total Universal Human Reference (UHR) RNA. For “GSC-SMARTer “, libraries were also generated from 10 ng total RNA.

**FIGURE 5:**
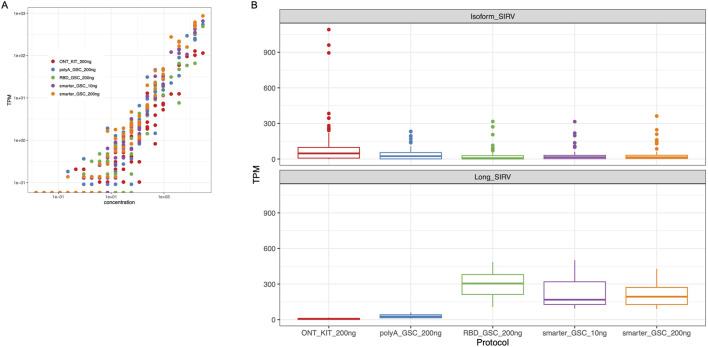
Validation of expression quantification accuracy. **(A)** Log-log plots of observed versus expected ERCC RNAs. Dots represent amounts of individual spike-in RNAs. **(B)** TPM Measurement of Isoform SIRV levels (upper panel) and long SIRV levels. TPM measurement assumes individual reads represent RNA molecules without transcript length correction.

The higher representation of full-length transcripts in the ONT kit could be explained by the unique combination of two features of the protocol: ligation of the adapter at the 3′-end of the polyA tail and priming of the cDNA synthesis from the distal end of the polyA tail on one hand, and attachment of the 5′ adaptor via a strand-switch mechanism on the other. The strand switching capacity is known to be greatly enhanced by the 5′-cap structure, which together with the 3′ distal start of cDNA synthesis may enrich for full-length transcripts (Wulf MG et al., 2019; Wulf MG et al., 2022). Random priming of the first cDNA synthesis is common to all three of the other protocols. Internal priming can, therefore, occur even though this should be limited via strand-displacement from downstream priming. The other protocol that has the 5′-end strand-switch feature is the SMARTer_GSC protocol but this approach is based on 3′-end random priming. Assessment of 5′-3′ gene body coverage of the top 1000 highly expressed genes (Figure 4A) and 5′-3′ ratios of the coverage of distal ends (Figure 4B) is at least partly consistent with these explanations. Specifically, the ONT-kit displayed the most uniform end-to-end coverage for transcript sizes up to 2 kb. For 2–20 kb size range, the ONT-kit increasingly showed 3′-end bias. Consistent with the strand-switch feature at the 5′end and random priming at the 3′-end, the SMARTer protocol displayed higher coverage at the 5′-end, displaying increasingly more severe 5′-end bias with increased transcript size. The RBD_GSC protocol was the most consistent across transcript sizes with persistent less coverage of the 50–100 bp at the very 5′-and 3′-ends. The PolyA_GSC protocol showed a similar trend to RBD_GSC except for transcripts of >5 kb where there was progressive but modest bias toward the 3′end, consistent with the protocol being polyA-based and the possibility that more opportunity for degradation exists in the case of longer transcripts. Supplementary Figure 2 shows an example of a large transcript (∼26 kb long) as visualized via IGV where the read distribution displayed the strong 3′-end bias of the ONT-kit protocol. Another difference between the protocols that may have affected these results is the number of beads-based purifications involved as depicted in Figure 1.

### Expression accuracy and dynamics, isoform detection, and variability

We next assessed the ability of the protocols to represent the expression levels of transcripts of varying abundance. To provide a “ground truth” for transcript abundance, we exploited the synthetic SIRV-Set 4 RNAs that were spiked into the UHR total RNA before library preparation. These have known sequences and predetermined amounts. They are subjected to the same steps of library preparation and sequencing as the endogenous UHR RNAs and their status is discerned only at the bioinformatics level after sequencing. SIRV-Set four is comprised of 92 ERCC synthetic RNAs with non-overlapping sequences at various concentrations that are ∼0.2–2 kb long, 69 SIRV isoform RNAs derived from seven model genes at the same concentration that are ∼0.2–2.5 kb long, and 15 equimolar long-SIRV RNAs with non-overlapping sequences that are 4–12 kb long (https://www.lexogen.com/sirvs/sirv-sets/). Thus, the ERCC RNAs allow assessment of dynamic range of expression levels, the SIRV isoform set is meant to represent transcriptome complexity better in terms of splice isoforms, and the long SIRVs mimic the range of average sizes of eukaryotic protein coding genes (Piovesan et al., 2019).

The performance of the four protocols in detecting ERCC RNAs was comparable, representing 50%–67% of the ERCC RNAs with the SMARTer protocol displaying the highest sensitivity (60%–67%). We compared the observed levels in the nanopore sequencing data to the expected yield of ERCC levels for comparison of the accuracy of the measurements, and we found that all four protocols displayed high ERCC correlation values (r > 0.86) with SMARTer-GSC protocol exhibiting higher correlation values (*0.94–0.96) as* compared to the other protocols *(0.86–0.90*) (Figure 5A).

The SIRV isoform analysis demonstrated that all four protocols performed similarly well in representing the various isoforms for a given detectable SIRV model gene locus. However, the ONT kit displayed the most variability in SIRV isoform measurements (Figure 5B upper panel). In particular, there were three outlier isoforms (SIRV 602, 609 and 615) that were measured at very high levels (35 to 43-fold higher than median expression value) in the libraries that were generated using the ONT kit (Supplementary Figure 3); all of which were from the same SIRV model locus (SIRV 6).

The detection and coverage of long SIRVs was the poorest for the ONT_kit, followed by the PolyA_GSC. The rRNA depletion protocols performed better (Figure 5B lower panel; Supplementary Figure 4), consistent with what was observed for endogenous UHR long transcripts (Supplementary Figure 2).

Although producing longer reads, the ONT long-read platform has typically produced reads with less overall sequence accuracy than short read platforms. This accuracy issue did not seem to hamper the sensitivity of detection of transcripts and splice variant profiling, for example, as evidenced by “ground truth” assessments we presented in the form of the data on ERRCs and SIRVs. In addition, iterative advancements in pore biophysical chemistry and base calling (Fu et al., 2019; Dohm et al., 2020; Sahlin and Medvedev, 2021; Ni et al., 2023; Kumari et al., 2024; Safar et al., 2024) are continually improving the accuracy for which our library construction method will be able to take advantage of. Of note, our approaches can also be adapted to other long-read platforms such as the PacBio technology.

In conclusion, this study demonstrates the adaptation of rRNA depletion protocols for ONT long read sequencing thereby presenting the opportunity to characterise polyadenylated and non-polyadenylated transcripts. These protocols do allow long read coverage of transcripts of various sizes and, as such, present substantial improvements over short read applications. A major area of improvement for these protocols is to render the resulting reads better at representing end-to-end spanning of full-length transcripts.

## Data Availability

The original data presented in the study is publicly available at National Center for Biotechnology Information’s Sequence Read Archive (identifier: PRJNA1186217). The data can be found at the following link: www.ncbi.nlm.nih.gov/bioproject/PRJNA1186217.
