# Supplementary material for: Adaptable and comprehensive approaches for long-read nanopore sequencing of polyadenylated and non-polyadenylated RNAs
Source: Front Genet. 2024 Dec 2;15:1466338. doi: 10.3389/fgene.2024.1466338 (PMC11647301; doi:10.3389/fgene.2024.1466338)
Supplement: Supplementary file 1 [file DataSheet2.pdf]

Supplementary Figure 1

Read length versus sizing of libraries using Agilent Bioanalyzer. (A) Mean Read length. (B) Size profiles on Agilent Bioanalyzer. (C) Average size based on values from Agilent Bioanalyzer data. (D) Percentage of fragments that are less than 500bp based on Agilent Bioanalyzer data. All libraries were generated from 200 ng total Universal Human Reference (UHR) RNA. For "GSC-SMARTer ", libraries were also generated from 10 ng total RNA.

Supplementary Figure 2

An Integrative Genomics Viewer image of the genomic region spanning the *CELSR2* gene. Genomic location-specific read depth ranges are indicated within each plot. All libraries were generated from 200 ng total Universal Human Reference (UHR) RNA. For "GSC-SMARTer ", libraries were also generated from 10 ng total RNA.

Supplementary Figure 3

Variability of isoform quantification using the ONT kit. An Integrative Genomics Viewer image of *SIRV6.* Location-specific read depth ranges are indicated within each plot. The models of the 13 isoforms corresponding to *SIRV6* are shown at the bottom. Of those the outliers are highlighted with red boxes. All libraries were generated from 200 ng total Universal Human Reference (UHR) RNA. For "GSC-SMARTer ", libraries were also generated from 10 ng total RNA.

Supplementary Figure 4

Detection and coverage of a representative of long SIRVs. An Integrative Genomics Viewer image of *SIRV12002.* Location-specific read depth ranges are indicated within each plot. All libraries were generated from 200 ng total Universal Human Reference (UHR) RNA. For "GSC-SMARTer ", libraries were also generated from 10 ng total RNA.

PCR-enriched Library Construction for Nanopore Sequencing

I. References

Reference Title	Reference Number
PCR-cDNA Barcoding Kit	SQK-PCB111.24

II. Materials and Equipment

Name	Supplier	Number: #	Model or Catalogue #	
NEB Paired-End Sample Prep Premix Kit – End Repair	NEB	E6875B-GSC		✓
NEB Paired-End Sample Prep Premix Kit – A Tail	NEB	E6876B-GSC		✓
NEB Paired-End Sample Prep Premix Kit – Ligation	NEB	E6877B-GSC		✓
LongAmp Hot Start *Taq* 2X Master Mix	NEB	M0533L		✓
PCR-cDNA Barcoding Kit	Oxford Nanopore	SQK-PCB111.24		✓
Fisherbrand Textured Nitrile gloves – various sizes	Fisher	270-058-53		✓
Ice bucket	Fisher	11-676-36		✓
Wet ice	In house	N/A	N/A	N/A
DNA *AWAY*	Molecular BioProducts	21-236-28		✓
AB1000 Plates	Thermo Scientific	SP-5201/150		✓
Gilson P2 pipetman	Mandel	GF-44801	✓	
Gilson P10 pipetman	Mandel	GF-44802	✓	
Gilson P20 pipetman	Mandel	GF23600	✓	
Gilson P200 pipetman	Mandel	GF-23601	✓	
Gilson P1000 pipetman	Mandel	GF-23602	✓	
Diamond Filter tips DFL10	Mandel Scientific	GF -F171203		✓
Diamond Filter tips DF30	Mandel Scientific	GF-F171303		✓
Diamond Filter tips DF200	Mandel Scientific	GF-F171503		✓
Diamond Filter tips DF1000	Mandel Scientific	GF-F171703		✓
Galaxy mini-centrifuge	VWR	37000-700	✓	
VX-100 Vortex Mixer	Rose Scientific	S-0100	✓	
Black ink permanent marker pen	VWR	52877-310		✓
Clear Tape Sealer	Qiagen	19570		✓
Aluminum Foils seals	VWR	60941-126		✓
Aluminum foil tape, 3"x 60 yds	Scotch/3M	34000740		✓
Eppendorf BenchTop Refrigerated Centrifuge 5810R	Eppendorf	5810 R	✓	
Bench Coat (Bench Protection Paper)	Fisher	12-007-186		✓
Small Autoclave waste bags 10"X15"	Fisher	01-826-4		✓
Anhydrous Ethyl Alcohol (100% Ethanol)	Commercial Alcohols	00023878		✓
IKA Works Vortexer	Agilent	MS2S9-5065-4428	✓	
22R Microfuge Centrifuge	Beckman	22R Centrifuge	✓	
PCRMax Alpha Cycler 4	Froggabio/Cole Parmer	AC496	✓	
Microlab NIMBUS	NIMBUS	Hamilton	✓	
Eppendorf Benchtop Centrifuge	Eppendorf	5810 R	✓	
70% Ethanol	In house	N/A	N/A	N/A
Qiagen Buffer EB – 250 mL	Qiagen	19086		✓
Ultrapure Water	Invitrogen	10977-023		✓
96 Low Profile Reservoir, Pyramid bottom	Thomas Scientific	1149J14		✓
Ampure XP Beads, 450 mL	Agencourt	A63882		✓
PCR Clean DX (ALINE beads)	ALINE Biosciences	C-1003-450		✓
USER Enzyme	NEB	M5505L		✓
MagMax express 96 Deep Well plates	Applied Biosystems	4388476		✓
ABgene Storage Plate 96-well, 1.2 mL square well, U-	Thermo Scientific	AB1127		✓

Name	Supplier	Number: #	Model or Catalogue #	
bottomed				
Adhesive foil -96 ONE TAB NS CS100).	VWR	60941-126		✓
ALPS 50V Microplate Heat Sealer	Thermo Scientific	AB-1443	✓	
EZPierce 20 um Thermal foil	ThermoFisher	AB1720		✓
NTSV adapter	IDT	N/A	Custom, see sequence below	N/A
MagLevitator	In House	N/A	N/A	N/A

NTSV: /5Phos/GATCGGAAGAGAAGATAGAGCGACAGGCAAGT TTTCTGTTGGTGCTGATATTGCTCTTCCGATC*T

*** is a phosphorothioate bond**

III. Introduction and Guidelines

1. General Guidelines

.

1.1. **MagLevitator should be kept plugged into the laptop at all times. It should also be left at the far right end of the deck when not in use. If it is not, follow instructions listed below for setup.**

Nimbus Set-up with MagLevitator

1.1.1. Setting up the MagLevitator should take place before running any protocol as the protocol will initialize it before showing the deck layout.

1.1.2. An error message will appear if the MagLevitator is not connected to the laptop or does not have power (e.g. surge protector accidentally turned off).

1.1.3. If this happens, stop & close the protocol, connect the MagLevitator and run the protocol again.

1.1.4. Place the MagLevitator on the far right end of the Nimbus deck.

1.1.5. For the next 2 steps, ensure the cables are to the right of the Nimbus pod gantry so it does not pull on the cords when the pod moves to the left.

1.1.6. Connect the USB cable into the back USB port (if present) on the laptop and connect it to the MagLevitator.

1.1.7. Connect the power cord to the MagLevitator.

1.1.8. Log onto the Nimbus and select program. From this point forward, you will be prompted with regards to any actions you will need to perform.

1.1.9. When prompted for the deck layout, move the MagLevitator to its position.

1.1.10. Ensure the electrical wires are not under any other pedestals!

1.1.11. Setup the Nimbus deck as per the prompted deck layout.

2. General Plate Guidelines

2.1. To avoid cross-well contamination, reaction plates should never be vortexed and plate seals should never be re-used. Use Nimbus for mixing.

2.2. Use only VWR foil seals for both short term storage and tetrad incubations, Adhesive foil EZPierce 20 µM Thermal Foil (Cat. No. AB1720) for UNG digestion/PCR, and 3M aluminum foil seal for long term storage.

2.3. After completion of every incubation step, quick spin the plate(s) at 4ºC for 1 minute at 2000g.

2.4. Sample plates can be stored at -20ºC overnight after every step except post "A" addition. **"A" addition and adapter ligation reactions must be performed on the same day.**

2.5. The reaction plates should be placed on ice throughout the day when not being worked on.

3. Positive and Negative Controls

3.1. The positive control template to be used for this protocol is UHR cDNA. The yield of library products constructed from the positive control is expected to differ from those of collaborators' samples. However, the yield should not differ significantly from that of previously constructed positive control.

3.2. The negative control template to be used for this protocol is Qiagen Elution Buffer. This control will ensure the absence of background products that result from the library construction process.

4. General Brew Preparation Guidelines

4.1. Double check the QA release and expiry date of each reagent and enzyme.

4.2. Thaw required reagents and place them on ice. Enzymes should be left in the freezer until ready to use. Each premix tube can be freeze thawed three times.

4.3. Reagents and enzymes should be well mixed, the former by pulse-vortexing and the latter by gentle flicking. After mixing, quick spin down in a mini-centrifuge.

4.4. All premixed and prepared brews should be well mixed by gentle, repeated pulse-vortexing to ensure equal distribution of all components and thus uniformity of enzymatic reactions across a plate. The end-repair and ligation brews are particularly viscous.

5. Nimbus Handling Guidelines

5.1. The Nimbus adds cDNA to the brew plate and it is therefore crucial that the required brew volume is accurately pre-dispensed by the technician (there should not be any dead volume). However, a dead volume is required for the indexing primer plate (5 µL/well).

5.2. The dead volume required by the Nimbus in the 96-well reservoir is 25 mL.

5.3. Confirm that the plate and tip box locations on the Nimbus deck match the software deck layout on the computer screen.

5.4. Ensure that plate seals are removed before starting the Nimbus program.

6. General notes on Nimbus programs

Note: If you are unsure of which Nimbus protocol version to use, please consult your supervisor.

The following steps are generally followed:

A. Start Hamilton Run control

B. Open File/Production/LibraryConstruction/LibraryConstruction-Scheduler.wfl3
*Note that file type must have the.wfl extension.

C. Select the correct starting material/library type: Nanopore_cDNA

The Nimbus bead cleanup modules employed in this SOP are based on the following conditions:

Bead Binding Time (mins)	1st Magnet Clearing Time (mins)	2 X 70% EtOH Wash Vol (µL)	Ethanol Air-dry Time (mins)	Elution Volume (µL)	Elution time (mins)	2nd Magnet Clearing time (mins)
15	7	150	5	10-25	3	2

Ethanol and beads must be warmed to room temperature for at least 30 minutes prior to use.

IV. Procedure

Note: ALINE beads (PCR Clean DX) and Ampure XP beads can be used interchangeably in the magnetic bead clean up steps.

1. End-Repair and Phosphorylation Reaction

1.1. The volume requirement for 1 reaction set up is as follows:

Solution	1 rxn (µL)
cDNA	35

Solution	1 rxn (µL)
End Repair Premix	23.5
**Reaction volume**	**58.5**

1.2. Log into the Nimbus program as follows:

Nimbus: File > Production> LibraryConstruction > Library Construction-Scheduler.wfl3> Nanopore_ss-cDNA >**End Repair**

1.3. The brew plate is the "REACTION" and the DNA plate is the "DNA Sample." After Nimbus program completion, seal the plates and quick spin at 4ºC for 1 minute. Inspect the reaction plates for any variations in volume.

1.4. Incubate End-Repair reaction plate at 20ºC for 30 minutes. The total reaction volume is 58.5 µL.

Tetrad Program: Run > LIBCOR > ER

2. Magnetic Bead Clean Up after End-Repair

2.1. Log into the following Nimbus program:

Nimbus: File > Production> LibraryConstruction > Library Construction-Scheduler.wfl3> Nanopore_ss-cDNA > **Bead Clean E.R.**

Note: Bead Clean E.R. ratio is 1:1 bead: sample ratio.

End-repaired product can be stored at -20ºC after the bead cleanup.

3. Addition of an 'A' Base (A-Tailing) Reaction

3.1. The volume requirement for 1 reaction set up is as follows:

Solution	1 rxn (µL)
End-Repair + BC cDNA	15
Adenylation Brew	10
**Reaction volume**	**25**

3.2. Log into the following Nimbus program:

Nimbus: File > Production> LibraryConstruction > Library Construction-Scheduler.wfl3> Nanopore_ss-cDNA > **A-tailing**

3.3. The brew plate is the "REACTION" and the DNA plate is the "DNA Sample." After Nimbus program completion, seal the plates and quick spin at 4ºC for 1 minute. Inspect the

reaction plates for any variations in volume.

3.4. Incubate A-tailed reaction plate at 37ºC for 30 minutes; 70ºC for 5 minutes; 4ºC for 5 minutes, hold at 4ºC.

Tetrad Program: Run > LIBCOR > ATAIL

3.5. After the incubation, store the template temporarily on ice. **This is NOT a safe stopping point.** Quick spin plate and store on ice while setting up the ligation reaction.

3.6. **Adenylated products are not bead cleaned prior to ligation.**

4. Nanopore 1st Adapter Ligation Reaction

4.1. Thaw the adapter stock aliquot in the laminar flow hood and immediately place on ice.

4.2. For 5th floor set up: adapter ligation brew (minus the adapter) must be made in the PCR Clean Room laminar flow hood on the 5th floor (room 510). Addition of adapter to the brew can be done on the bench.

For 6th floor set up: adapter ligation brew (minus the adapter) must be made in the laminar flow hood. Addition of adapter to the brew must be done on the bench.

4.3. The volume requirement for 1 reaction set up is as follows:

.

Solution	1 rxn (µL)
Adenylated template	25
2X Ligation Premix	12.5
Nanopore Adapter (NTSV) 10nM	0.5
UltraPure Water	4.5
**Reaction volume**	**42.5**

4.4. Generate the ligation Brew Mix calculator using LIMS:

LIMS: Mix Standard Solutions > **cDNA_Nanopore_Ligation**> *follow the prompts* > Save Standard Solution

To minimize adapter-adapter ligation, work quickly on ice and proceed as follows:

4.4.1. Prepare the ligation brew in an appropriate sized tube according to the chemistry calculator.

4.4.2. Add the adapter to the brew last, not more than 10 min before brew addition on Nimbus. Make sure the brew is on ice at all times.

4.4.3. Dispense the appropriate amount (17.5 µL) of brew into an AB1000 plate.

4.4.4. Cover the brew plate with plate seal and quick spin at 4ºC for 1 minute.

4.4.5. Keep plates on ice but *proceed quickly* to the next step.

4.4.6. Log into the following Nimbus program:

Nimbus: File > Production> LibraryConstruction > Library Construction-Scheduler.wfl3> Nanopore_ss-cDNA > **Adapter Ligation**

4.4.7. The brew plate is the "REACTION" and the cDNA plate is the "DNA Sample." After Nimbus program completion, seal the plate and quick spin at 4ºC for 1 minute. Inspect the reaction plate for any variations in volume.

4.4.8. Incubate Ligation reaction plate at 20ºC for 15 minutes

Tetrad Program: LIBCOR> LIGATION

5. Magnetic Bead Clean Up after Adapter Ligation

5.1. The input volume for this step is 42.5 µL per well.

5.2. Log into the following Nimbus program:

Nimbus: File > Production> LibraryConstruction > Library Construction-Scheduler.wfl3> Nanopore_ss-cDNA > **Bead clean Ligation (1x)**

Note that Note: Bead Clean ratio is 0.8:1 bead: sample ratio and sample will be eluted in 24 µL of EB.

cDNA can be stored at -20°C after the bead cleanup.

6. USER digestion

6.1. The volume requirement for 1 reaction set up is as follows:

Solution	1 rxn (µL)
Ligated cDNA	23.5
USER	1.5
**Reaction volume**	**25**

6.2. Log into the Nimbus program as follows:

Nimbus: File > Production> LibraryConstruction > Library Construction-Scheduler.wfl3> Nanopore_ss-cDNA >**USER Digestion**

6.3. The brew plate is the "REACTION" and the DNA plate is the "DNA Sample." After Nimbus program completion, seal the plates and quick spin at 4ºC for 1 minute. Inspect the reaction plates for any variations in volume.

6.4. Incubate USER reaction plate at 37ºC for 15 minutes; 98ºC for 1 minute.

Tetrad Program: Run > LIBCOR >LC_Nanopore_USER

7. Magnetic Bead Clean Up after USER Digestion

7.1. Log into the following Nimbus program:

Nimbus: File > Production> LibraryConstruction > Library Construction-Scheduler.wfl3> Nanopore_ss-cDNA > **Bead Clean USER digestion**

Note: Bead Clean ratio is 1:1 bead: sample ratio and sample will be eluted in 11 µL of EB.

cDNA can be stored at -20ºC after the bead cleanup.

8. Indexed PCR (iPCR) Amplification

8.1. Thaw the Indexing Primer Plate in a working bench across from Nimbus on the 5th floor or in the 6th floor Library Construction Room, quick spin at 4ºC for 1 minute and immediately place on ice.

8.2. To keep track of freeze-thaw cycles, mark off the indexing primer plate each time the plate is thawed even if it is not used.

8.3. The maximum freeze-thaw cycles for the indexing primer plate are **5 times.**

8.4. Ensure there is enough volume including the Nimbus dead volume. Inspect the thawed index primer plate after spin down to ensure there are no cracked wells.

8.5. iPCR brew (minus the primers) must be made in the PCR Clean Room laminar flow hood on the 5th floor (room 510) or the hood in the Library Construction Room. Addition of the Indexing Primer is performed by the Nimbus.

8.6. The volume requirement for 1 reaction set up is as follows:

Solution	1 rxn (µL)
Adapter Ligated + BC DNA	10
LongAmp Hot Start *Taq* 2X Master Mix	12.5
Index Primers	2
**Reaction volume**	**24.5**

8.7. Dispense 12.5 µL of LongAmp Hot Start *Taq* 2X Master Mix into an AB1000 plate. Cover with plate seal and quick spin at 4°C for 1 minute.

8.8. Log into the following Nimbus program:

Nimbus: File > Production> LibraryConstruction > Library Construction-Scheduler.wfl3> Nanopore ss-cDNA>**Index PCR**

8.9. The Nimbus program for iPCR setup for is as follows:

8.9.1. Addition of index primers to the DNA Source Plate (post BC ligation plate)

8.9.2. Transfer of DNA+ index primer to the brew plate.

8.10. After Nimbus program completion, seal the plate and quick spin at 4ºC for 1 minute. Inspect the reaction plate for any variations in volume.

*Heat seal the plate using Adhesive foil EZPierce 20 µm Thermal foil (Cat. No. AB1720, Thermo Fisher). The equipment used for this is ALPS 50V Microplate Heat Sealer (Cat. No. AB-1443, Thermo Scientific). Please see Appendix B for instructions.

8.11. Run the "Nano_cDNA_PCR13" PCR program. Use a rubber pad on top of the reaction plate.

PCR parameters:

- 95˚C 30 sec
- 95˚C 15 sec
- 56˚C 15 sec
- 65˚C 3 min
- 65˚C 6 min
- 4˚C ∞

13 cycles (95˚C 15 sec, 56˚C 15 sec, 65˚C 3 min)

9. Top up with Qiagen EB

9.1. Nimbus adds the 75 µL of Qiagen EB to bring the total volume of 100 µL.

9.2. Log into the following Nimbus program:

Nimbus: File > Production> LibraryConstruction > Library Construction-Scheduler.wfl3> Nanopore ss-cDNA > **Qiagen EB TopUp**

10. Post-PCR Size Selection

10.1. The input volume for this step is 100 µL per well. The PCR reaction is 25 µL total volume. The nimbus will add 75 µL of Qiagen of EB.

10.2. Log into the following Nimbus program:

Nimbus: File > Production> LibraryConstruction > Library Construction-Scheduler.wfl3> Nanopore ss-cDNA > **Bead clean iPCR (1x)**

10.3. Note that the EB for elution is from the Nanopore kit for this step. The final elution volume is 13 µL.

11. Preparation of Diluted Library QC Plate

11.1. Prepare a 10x dilution QC plate using the following Nimbus program:

Nimbus: File > Production> LibraryConstruction > Library Construction-Scheduler.wfl3> Nanopore ss-cDNA > **Dilute for QC**

The Nimbus will transfer 18 µL of Qiagen EB to a new plate and then transfer 2 µL of final library product to the EB plate. This 10x dilution will be used first for Quant-iT (2 µL) and the remaining 18 µL will subsequently be used for Caliper.

12. Quant-iT/Qubit QC

12.1. Refer to the following SOPs for setting up the QC plate prior to normalization/pooling:

LIBPR.0108 Automated DNA Quantification using the dsDNA Quant-iT High Sensitivity Assay Kit and VICTOR3V/VICTOR X3<br>or<br>LIBPR.0153 Quantifying DNA Samples using the Qubit 4 Fluorometer

12.2. For Quant-iT, use the 10x dilution plate or undiluted library as source plates for the QC. Log into the following Nimbus program:

Nimbus: File > Production> LibraryConstruction > Library Construction-Scheduler.wfl3> Nanopore ss-cDNA > **Quant-It**

12.3. For Qubit, use the undiluted DNA from post-library construction size selection.

13. HS DNA Agilent QC

LIBPR.0017 Operation and Maintenance of the Agilent 2100 Bioanalyzer for DNA samples

Send the results to the APC for approval.

14. Normalization on JANUS G3 (if necessary)

14.1. Refer to the following SOP for normalization on JANUS G3:

LIBPR.0146 JANUS G3 Normalization and Pooling of DNA Samples

15. Pooling Samples into 1.5 mL Tubes on JANUS G3 (if needed) or Rearray Unpooled Samples into 1.5 mL Tubes

15.1. Refer to the following SOP for pooling on JANUS G3:

LIBPR.0146 JANUS G3 Normalization and Pooling of DNA Samples

16. Qubit QC on Pooled Samples for submission

16.1. Refer to the following SOP:

LIBPR.0153 Quantifying DNA Samples using the Qubit 4 Fluorometer

16.2. The average base pair size obtained should exceed 1 kb and the total yield of the pooled libraries should exceed 30 ng (per 10 µL).

Appendix B: ALPS 50V Microplate Heat Sealer

NOTE: The seals should be stored in the foil seal packaging to maintain proper orientation. Failure to orient the foil seal with the adhesive side down in the plate sealer will result in the seal adhering to the instrument rather than the plate.

1. Turn on the ALPS 50V heat sealer and allow the instrument to warm up. The Heat on/off LED will flash during this time and stay on once the desired temperature is reached. The sealer should be pre-set for 165°C, 3 second seal time.

2. Place the foil seal on top of the input plate (shiny side up).

3. Place the plate on the plate carrier so that well A1 is in the back left corner. Avoid touching the heating surface while loading the sample plate to prevent injury.

4. Grasp the handle and lower to thermally compress the foil seal onto the input plate. Do NOT apply more pressure to the handle than necessary. When the correct pressure is achieved, an audible tone will sound and the timer will count down to zero.

5. Once the timer reaches zero, another audible tone will sound. Raise the handle to release the heater plate.

6. Rotate the plate so that well A1 is in the front right corner (H12 will be in the back left corner) and repeat the sealing steps 4 and 5.

7. Use a roller seal to ensure that all wells are properly sealed.

8. Put a thermal pad on top of the output plate, then close and tighten the lid.

Appendix C: Manual Library Construction

1. End Repair & Phosphorylation

Solution	1 rxn (µL)
DNA	35
NEB End Repair Premix	23.5
**Reaction volume**	**58.5**

1.1. Transfer 23.5 µL of NEB End Repair Premix into wells of a destination plate.

1.2. Transfer 35 µL of cDNA to End Repair Premix, mix using 80% volume, 10X.

1.3. Tetrad Program: LIBCOR>ER; 20ºC for 30 minutes; hold 4ºC.

1.4. Safe stopping point if stored at -20ºC.

2. Bead Clean End Repaired & Phosphorylated Template

Ethanol and Magnetic beads must be incubated at room temperature for at least 30 minutes before use.

DNA volume (µL)	Bead Volume (µL)	Mixing Volume (µL)	Bead Binding Time (mins)	Magnet Clearing Time (mins)	Supernatant Volume (µL)	2x 70% EtOH Wash Vol (µL)	Ethanol Air Dry Time (mins)	EB Elution Volume (µL)	Elution Time (mins)	Magnet Elution Time (mins)
58.5	58.5	93	15	7	117	150	5	16	3	2

2.1. Note: This is a safe stopping point. Do not proceed to adenylation unless you have adequate time to perform ligation reaction as well.

3. A-Tailing

Solution	1 rxn (µL)
End-Repair + BC DNA	15
NEB Adenylation Premix	10
**Reaction volume**	**25**

3.1. Transfer 10 µL of NEB Adenylation Premix to 15 µL of repaired/phosphorylated DNA.

3.2. Tetrad Program: LIBCOR>ATAIL (for 30 minutes; 70ºC for 5 minutes; 4ºC for 5 minutes, hold at 4ºC)

3.3. Proceed directly to in-tandem ligation (**do not bead clean after Adenylation**). Store on ice while preparing Ligation premix and adapters.

4. Adapter Ligation

Solution	1 rxn (µL)
Adenylated template	25
2X Ligation Premix	12.5
Nanopore Adapter (NTSV) 10nM	0.5
UltraPure Water	4.5
**Reaction volume**	**42.5**

Ligation calculator:

LIMS: Mix Standard Solutions > **cDNA_Nanopore_Ligation** > *follow the prompts* > Save Standard Solution

4.1.Transfer 17.5 µL of ligation brew to 25 µL of adenylated template.

4.2.Reset pipette to 80% total volume, mix 10X.

4.3.Select tetrad program: LIBCOR>LIGATION (20ºC for 15 minutes)

4.4.Set a timer for 15 minutes. Quick spin plate and store on ice immediately after the 15 minute ligation.

5. **Bead Clean Post-Ligation**

DNA volume (µL)	Bead Volume (µL)	Mixing Volume (µL)	Bead Binding Time (mins)	Magnet Clearing Time (mins)	Supernatant Volume (µL)	2x 70% EtOH Wash Vol (µL)	Ethanol Air Dry Time (mins)	EB Elution Volume (µL)	Elution Time (mins)	Magnet Elution Time (mins)
42.5	34	61	15	7	76.5	150	5	25	3	2

6. If the cDNA preparation included dUTP marking for second strand synthesis, proceed to Step 7; if not (e.g. cDNA from 10x Chromium single cell RNA run), proceed to step 8**.**

7. **USER Digestion**

Solution	1 rxn (µL)
Ligated cDNA	23.5
USER	1.5
**Reaction volume**	**25**

7.1.Transfer 1.5 µL of USER to 23.5 µL of adenylated template.

7.2.Reset pipette to 80% total volume, mix 10X.

7.3.Select tetrad program: LC_Nanopore_USER (37ºC for 15 minutes; 98ºC for 1 minute)

7.4.Quick spin plate and store on ice immediately after the incubation.

8. Bead Clean Post-USER digestion

DNA volume (µL)	Bead Volume (µL)	Mixing Volume (µL)	Bead Binding Time (mins)	Magnet Clearing Time (mins)	Supernatant Volume (µL)	2x 70% EtOH Wash Vol (µL)	Ethanol Air Dry Time (mins)	EB Elution Volume (µL)	Elution Time (mins)	Magnet Elution Time (mins)
25	25	40	15	7	50	150	5	11	3	2

8.1.The ligated template can be stored at -20˚C after the first or second bead clean up step.

9. PCR enrich adapter-ligated template

Solution	1 rxn (µL)
Adapter Ligated + BC DNA	10
LongAmp 2x Master Mix	12.5
Index Primers	2
**Reaction volume**	**24.5**

9.1. Run the "Nano_cDNA_PCR13" PCR program. Use a rubber pad on top of the reaction plate.

PCR parameters:

- 95˚C 30 sec
- 95˚C 15 sec
- 56˚C 15 sec
- 65˚C 3 min
- 65˚C 6 min
- 4˚C ∞

(95˚C 15 sec, 56˚C 15 sec, 65˚C 3 min: 13 of cycles)

9.2.Heat seal the plate using Adhesive foil EZPierce 20 µm Thermal foil (Cat. No. AB1720, Thermo Fisher). The equipment used for this is ALPS 50 V Microplate Heat Sealer (Cat. No. AB-1443, Thermo Scientific). Please see Appendix B for instructions.

9.3.PCR-enriched template can be stored at -20˚C or proceed immediately to bead clean PCR enriched template.

10. Bead Clean post iPCR

- Top up PCR reaction with 75 µL of Qiagen EB.

- The input volume for this step is 100 µL per well.

DNA volume (µL)	Bead Volume (µL)	Mixing Volume (µL)	Bead Binding Time (mins)	Magnet Clearing Time (mins)	Supernatant Volume (µL)	2x 70% EtOH Wash Vol (µL)	Ethanol Air Dry Time (mins)	Nanopore EB Elution Volume (µL)	Elution Time (mins)	Magnet Elution Time (mins)
100	70	136	15	7	170	150	5	13	3	2

Note that the EB for the final elution is from the Nanopore kit for this step.
Library can be stored at -20ºC after the bead clean up post PCR.

11. QC Final Library Products

11.1. Run 1 µL of each final library product on Agilent DNA HS chip assay.

11.2. Quantify each final library product by Qubit HS DNA assay using 1 µL of the library.

11.3. If required, normalize and pool samples manually and quantify pool by Qubit HS DNA assay.

11.4. The average base pair size obtained should exceed 1kb and the total yield of the pooled libraries should exceed 30 ng (per 10 µL).
