# Supplementary figures and images for: Adaptable and comprehensive approaches for long-read nanopore sequencing of polyadenylated and non-polyadenylated RNAs
Source: Front Genet. 2024 Dec 2;15:1466338. doi: 10.3389/fgene.2024.1466338 (PMC11647301; doi:10.3389/fgene.2024.1466338)

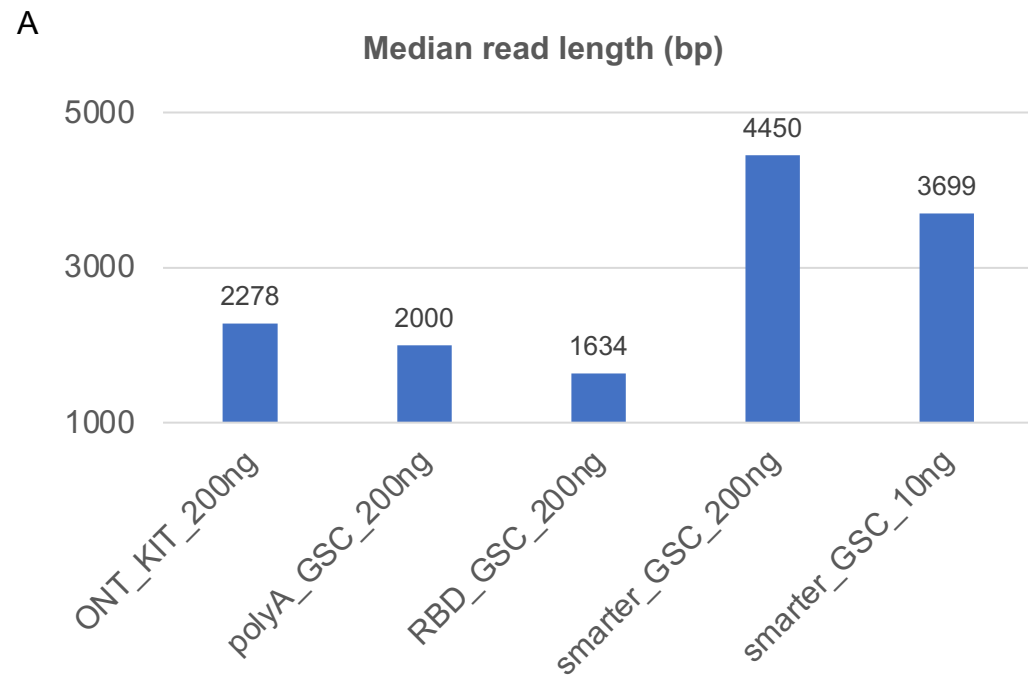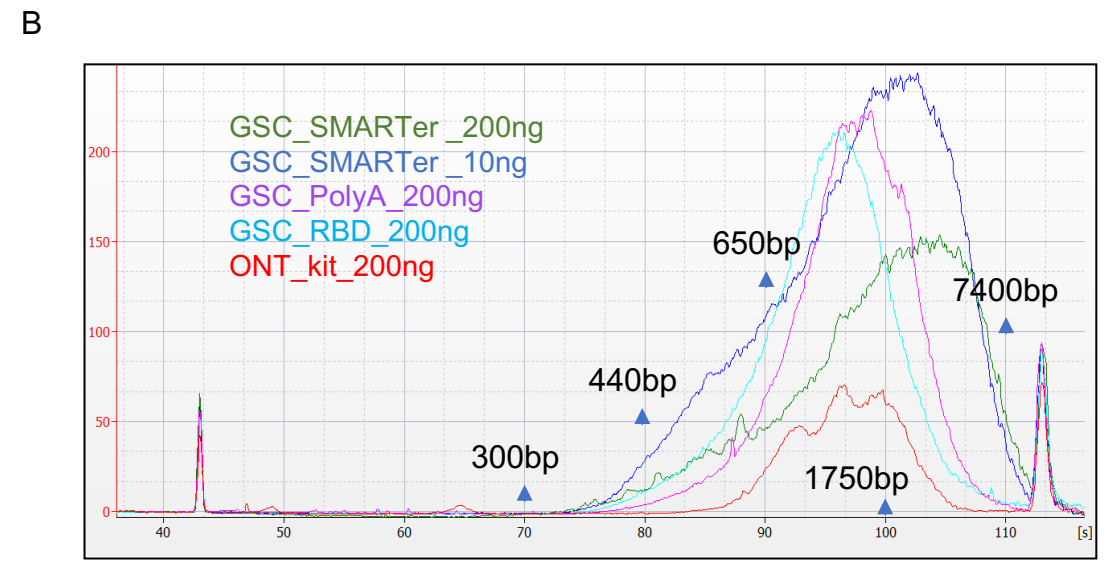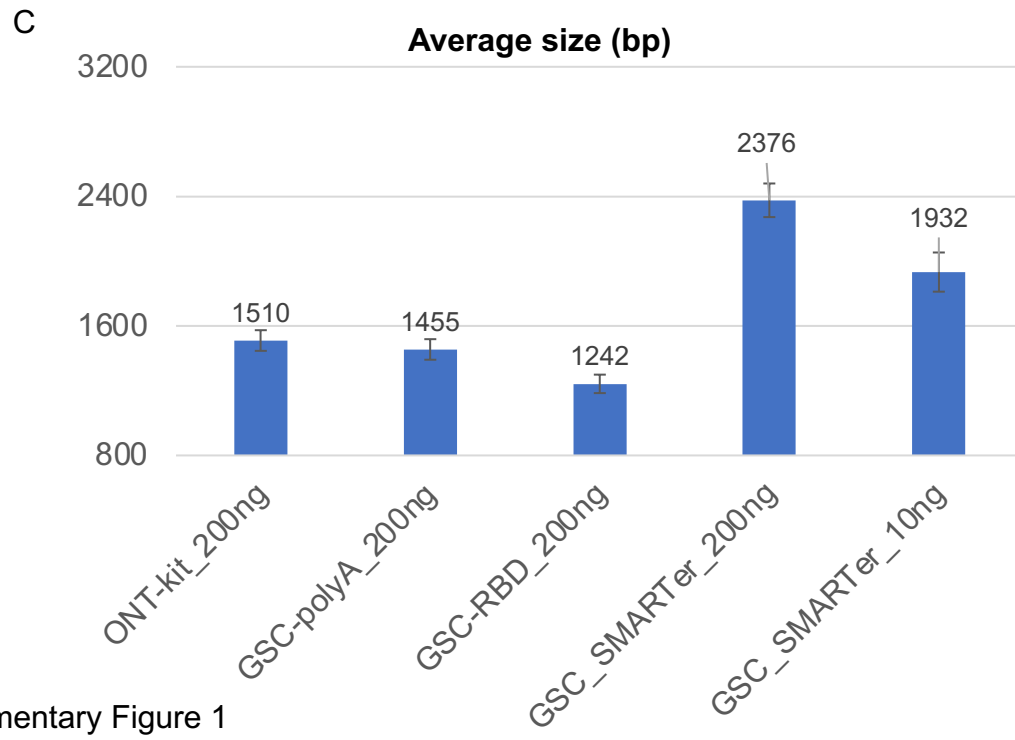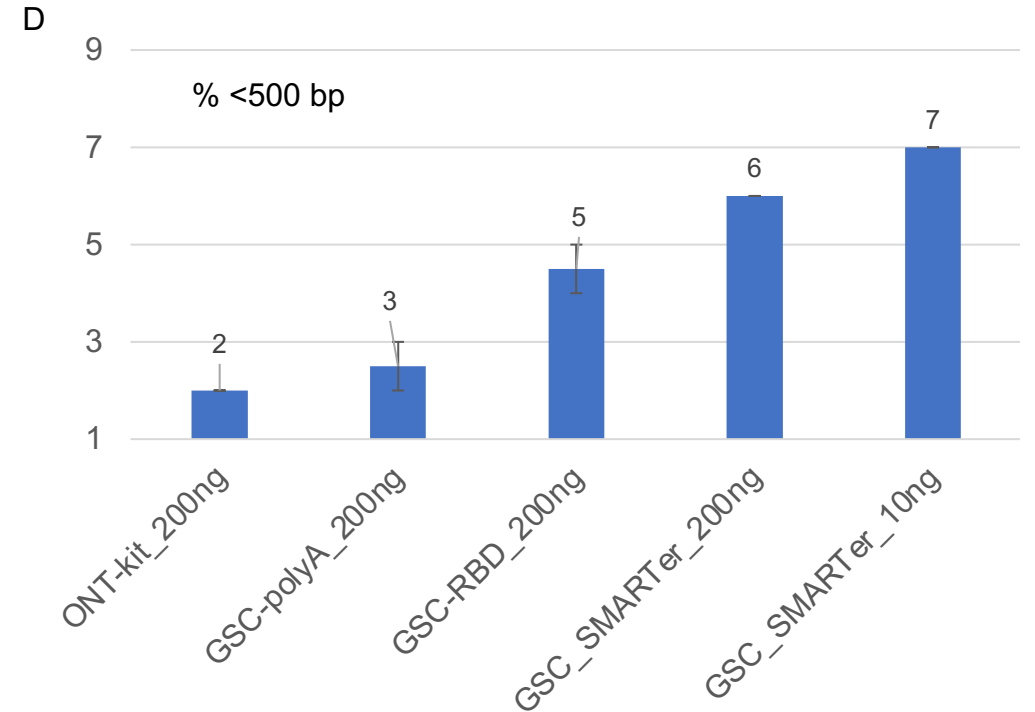

Supplementary Figure 1

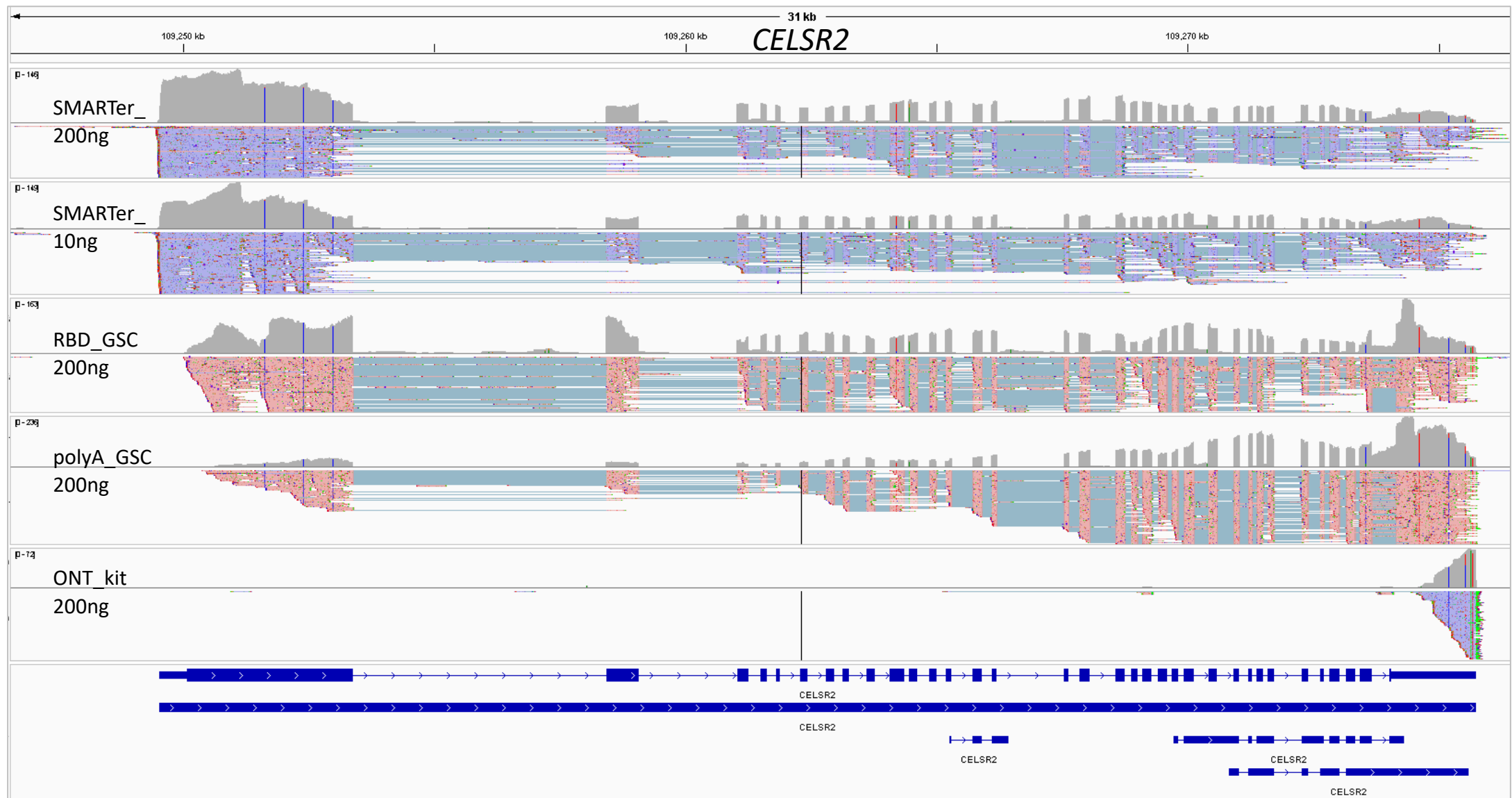

Supplementary Figure 2

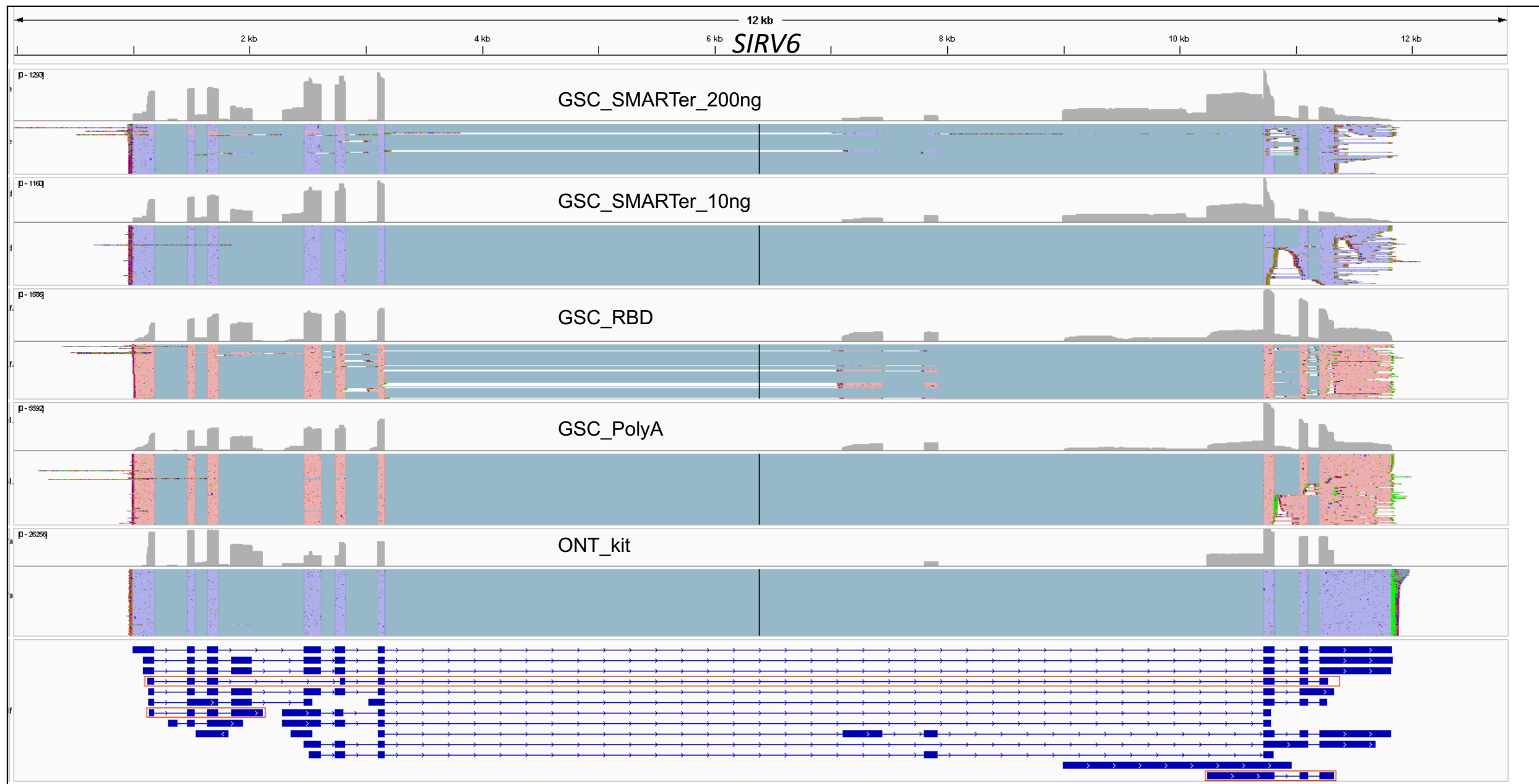

Supplementary Figure 3

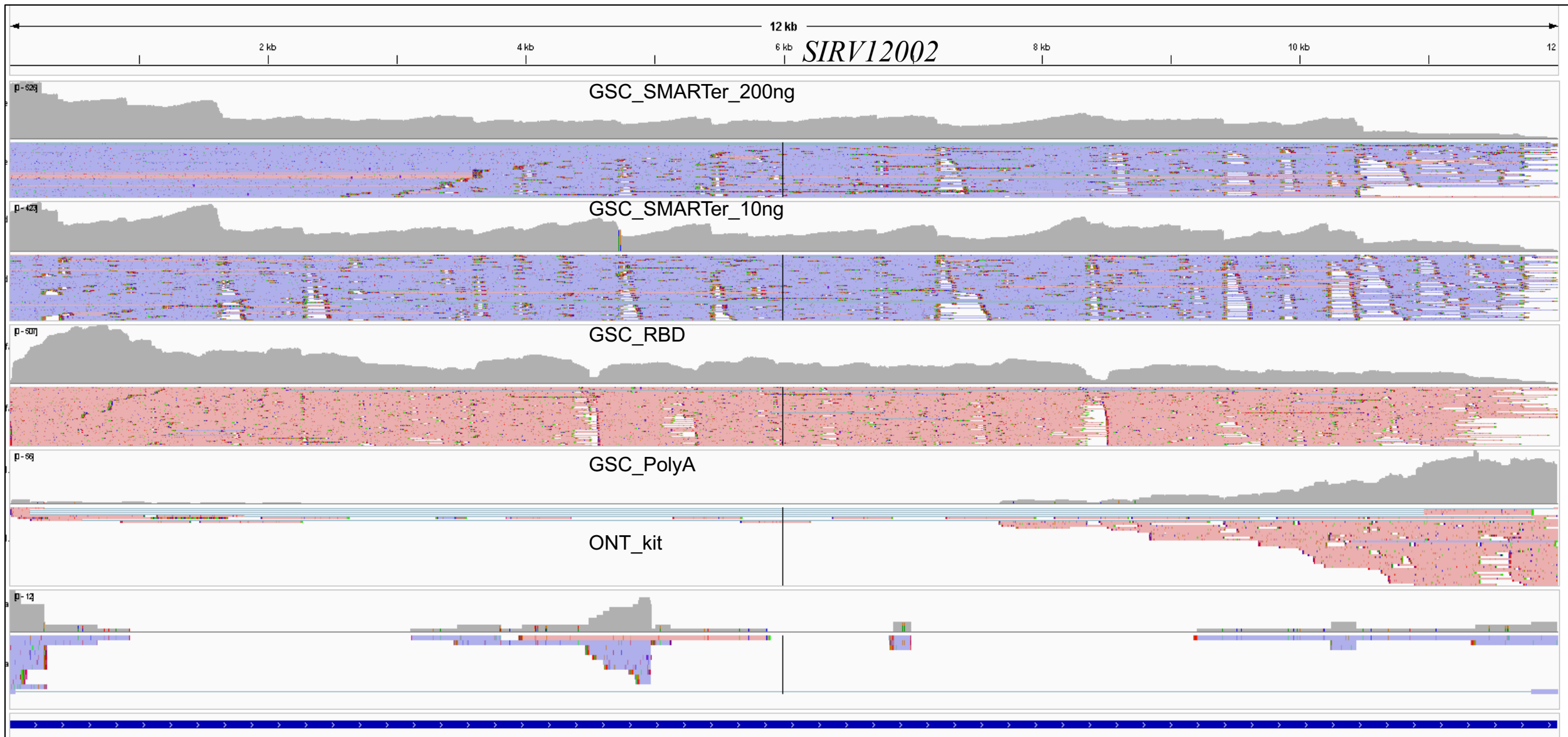

Supplementary Figure 4

Supplement: Supplementary file 2 [file DataSheet1.pdf]
